# Immunotherapy as a treatment modality for mucosal melanoma of the head and neck: A systematic review

**DOI:** 10.1097/MD.0000000000029979

**Published:** 2022-08-05

**Authors:** Jad Wehbe, Dominic Jaikaransingh, Abigail Walker

**Affiliations:** a St George’s University of London, – Chelsea and Wesminster Hospital, London, United Kingdom; b St George’s University of London, St George’s Hospital, London, United Kingdom; c University of Glasgow, Royal Brisbane Hospital, Australia.

**Keywords:** head and neck cancer, immunotherapy, mucosal melanoma, survival rates

## Abstract

**Introduction:**

Mucosal melanoma (MM) is a rare disease, accounting for approximately 1.4% of all melanomas and only 0.03% of all new cancer diagnoses. Traditionally, it has been associated with a poor prognosis, with an overall 5-year survival rate of <25%. Progress in treatment has been hindered by its rarity and lack of evidence. However, studies on the treatment of subcutaneous melanoma with immunotherapy have demonstrated significant improvement in survival rates and have become a core part of oncological strategies. This paper discusses the revision of the evidence for the use of immunotherapy in the head and neck.

**Methods:**

This systematic review was conducted on January 19, 2019. The Medline and Embase databases were searched. In total, 509 articles were collated and screened. Inclusion criteria for the study included treatment-naive cohorts, cohorts with recurrent disease, primary outcomes with overall survival and disease-free survival at 5 years and at the longest follow-up, and studies of adults with MM in whom immunotherapy was reported as a treatment strategy. The exclusion criteria included duplicate papers, anatomical sites other than the head and neck, case reports, and those not published in English.

**Results:**

Fifty-two papers out of the 509 collated papers met the inclusion criteria. The results are shown as a comparison of yearly survival rates following different treatment modalities (immunotherapy vs nonimmunotherapy) at 2, 3, and 5 years. It was found that, with immunotherapy, survival rates at all intervals were higher than those without immunotherapy.

**Discussion:**

Immunotherapy outcomes in small studies have shown good data for increasing survival rates at yearly intervals in MM of the head and neck. Larger clinical trials are needed to accurately distinguish the efficacy and survival outcomes of immunotherapy when compared with treatment modalities, excluding immunotherapy. However, the ability to perform larger trials is limited by the rarity of MM of the head and neck.

**Key points**

- Mucosal melanoma of the head and neck has a poor prognosis. Structured treatment directed at these malignancies remains variable owing to the low incidence of the disease. Surgery, radiotherapy, and chemotherapy remain the mainstay of treatment. With limited literature available, immunotherapy demonstrates a promising aspect of treatment for prolonging survival rates in these malignancies. Further trials are necessary to confirm the efficacy of immunotherapy.

## 1. Introduction

Primary mucosal melanomas are rare, biologically aggressive neoplasms with poor outcomes. They account for 1.4% of all melanomas and only 0.3% of new cancer diagnoses.^[1]^ The distribution of head and neck, female genital tract, anal/rectal, and urinary tract sites was 55.4%, 18.0%, 23.8%, and 2.8%, respectively.^[2]^ The median age at presentation is the seventh decade, with a tendency for women to be affected more than men.^[2]^

In the head and neck region, there seems to be a predominance of the disease in the sinonasal region, accounting for 59% to 80% of cases.^[3]^ Mutations associated with mucosal melanoma are poorly understood. A paper by Nassar and Tan, published in 2020, looking at the mutational landscape of mucosal melanoma, showed, using targeted sequencing, whole-exome sequencing, and whole-genome sequencing, that the mutation is unknown in 44% of cases. However, SF3B1 was implicated in 15% of cases, KIT in 13%, NF1 in 14%, NRAS in 8%, and BRAF in 6%.^[4]^

The overall 5-year survival rate is poor, with 1 study citing a 21.7% rate in 695 patients.^[5]^ Treatment of mucosal melanoma has been subject to multiple trials, some of which include surgery alone, surgery with chemotherapy, surgery with radiotherapy, surgery with chemotherapy, and, finally, with or without immunotherapy. Surgery remains the primary therapeutic intervention given that complete resection is feasible in a set anatomical location. Treatment with immunotherapy is novel; thus, studies proving the efficacy of immunotherapy are lacking.

## 2. Methods

### 2.1. Literature search

A literature review was conducted by searching Medline and Embase, going back as far as the database went, until 2019. The databases were searched using the terms listed in Table 1. A total of 509 citations were collected.

**Table 1:** Search strategy.

2 (mucosal melanoma*).ti.ab
7 Exp “GENERAL SURGERY”/
8 (surger*).ti.ab
9 Exp “DRUG THERAPY”/
10 (chemotherapy).ti.ab
11 Exp RADIOTHERAPY
12 (radiotherapy OR “radiation therapy” OR RT).ti.ab
13 Exp IMMUNOTHERAPY
14 (immunotherapy).ti.ab
15 Exp “COMBINED MODALITY THERAPY”/
16 ((multimodality OR combin*) ADJ3(therapy OR treatment)).ti.ab
17 (7 OR 8 OR 9 OR 10 OR 11 OR 12 OR 13 OR 14 OR 15 OR 16)
18 exp MORTALITY/
19 (mortality).ti.ab
20 Exp RECURRENCE/
21 (recurrence*).ti.ab
22 Exp “DISEASE-FREE SURVIVAL”/
23 ((disease Or progression) ADJ3 free survival)
24 (18 OR 19 OR 20 OR 21 OR 22 OR 23)
25 (2 AND 17 AND 24)
26 (mucosal melanoma*).ti.ab
27 exp “GENERAL SURGERY”/
28 (surger*).ti.ab
29 exp “DRUG THERAPY”/
30 (chemotherapy).ti.ab
31 exp RADIOTHERAPY
32 (radiotherapy OR “radiation therapy” OR RT).ti.ab
33 exp IMMUNOTHERAPY/
34 (immunotherapy).ti.ab
35 exp “COMBINED MODALITY THERAPY”/
36 ((multimodality or combin*) ADJ3 (therapy OR treatment)).ti.ab
37 exp MORTALITY
38 (mortality).ti.ab
39 exp RECURRENCE
40 (recurrence*).ti.ab
41 exp “DISEASE-FREE SURVIVAL”/
42 ((disease OR progession)ADJ3 free survival).ti.ab
44 (37 OR 38 OR 39 OR 40 OR 41 OR 42)
45 (23 AND 43 AND 44)

### 2.2. Study selection

An initial primary screen was conducted by Authors JW and DJ to include only articles on human subjects, articles in English, and articles with full text availability. The primary screening included carefully reviewing the database for duplicates. Furthermore, the primary screening excluded articles with non–head and neck mucosal melanomas.

A second screen was performed to stratify articles according to anatomical site (sinonasal or all head and neck), whether they were case reports, whether treatment outcomes were reported, whether 1 treatment modality was used, and whether immunotherapy was used. In total, 52 articles met the inclusion criteria, which consisted of treatment-naive cohorts, those with recurrent disease, primary outcomes with overall survival and disease-free survival at 5 years, and at the longest follow-up, and studies of adults with mucosal melanoma in whom immunotherapy was reported as a treatment strategy.

**Figure 1.**
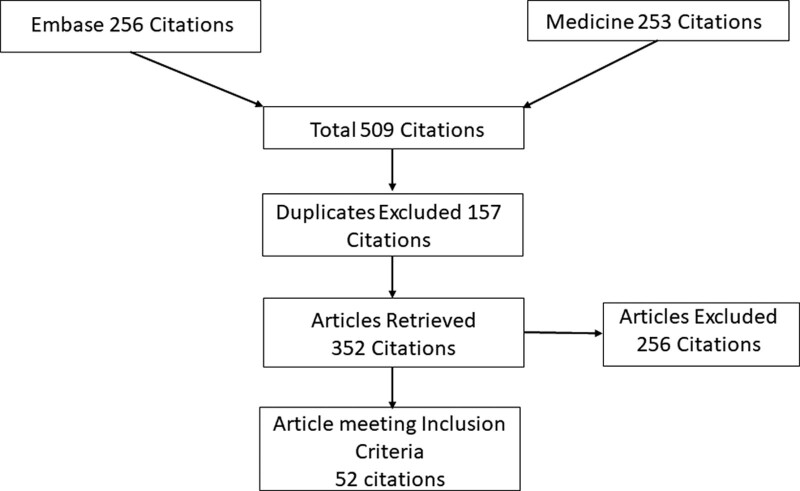
PRISMA chart showing inclusion and exclusion criteria. The above PRISMA chart demonstrates the inclusion and exclusion criteria for the study. The inclusion criteria included treatment-naive cohorts, those with recurrent disease, primary outcomes with overall survival and disease-free survival at 5 yr, and at longest follow-up, and studies of adults where immunotherapy was reported as a treatment modality. PRISMA = Preferred Reporting Items for Systematic reviews and Meta-Analyses.

A systematic review was conducted and reported in accordance with the Preferred Reporting Items for Systematic Reviews and Meta-Analyses flow chart (Fig. 1).

The characteristics of the included studies are shown in Table 2, outlining authors, country of study, number of patients in the study, treatment modalities, median time at follow-up, and histological type of cancer.

**Table 2:** Characteristics of included studies.

Study	Country	No. of patients	Treatment modalities	Median follow-up	Cancer type
Zhang 2018	China	162	Immunotherapy alone (n = 118)	Not reported	Mucosal melanoma (n = 41)
			Chemotherapy alone (n = 44)		Cutaneous melanoma (n = 121)
Namikawa 2018	Japan	30	2 immunotherapy agents (nivolumab + ipilimumab) every 3 wk for 4 doses, followed by biweekly nivolumab	14.1 mo (5.2–27.7)	Mucosal melanoma (n = 12)
					Nonacral cutaneous (n = 8)
					Acral cutaneous (n = 7)
					Uveal (n = 2)
					Unknown primary (n = 1)
Maxwell 2018	United States	1	Surgery + radiation +immunotherapy	Not applicable	Mucosal melanoma (n = 1)
Theirauf 2018	Germany	21	Surgery (n = 7)	51 mo (2–202 mo)	Mucosal melanoma (n = 21)
			Surgery + radiation (n = 9)		
			Surgery + interferon (n = 4)		
			Chemotherapy (n = 1)		
Kiyohara 2018	Japan	610	Immunotherapy (n = 610)	Not specified	Cutaneous (n = 389)
			Number of previous therapy (not specified)		Mucosal (n = 208)
			1 (n = 205)		Other (n = 50)
			2 (n = 116)		Unknown (n = 34)
			≥3 (n = 197)		
			Unknown (n = 162)		
Tsui 2018	Not specified	1	Surgery + radiotherapy + immunotherapy	Not applicable	Mucosal melanoma (n = 1)
Fujisawa 2017	Japan	60	Immunotherapy (n = 53)	Not specified	Acral lentiginous (n = 18)
			Radiotherapy + immunotherapy (n = 7)		Mucosal (n = 14)
					Nodular (n = 12)
					Superficial spreading (n = 8)
					Lentigo (n = 2)
					Others/unknown (n = 6)
Yamada 2017	Japan	38	Surgery (n = 16)	41.1 mo (1–137 mo)	Mucosal melanoma (n = 38)
			Surgery + immunotherapy (n = 3)		
			Surgery + chemotherapy + immunotherapy (n = 9)		
			Surgery + chemo (n = 9)		
			Surgery + chemoradiotherapy (n = 1)		
Liu 2017	China	51	Surgery (n = 48)	59.0 mo	Mucosal melanoma (n = 51)
			Radiotherapy (n = 33)		
			Chemotherapy (n = 10)		
			Immunotherapy (n = 13)		
Kuo 2017	Toronto	17	Immunotherapy (n = 17)	10.1 mo (0.8–56.6)	Mucosal melanoma (n = 17)
Shoushtari 2018	United States	81	Other modalities + immunotherapy (n = 20)	10.3 (0.5–90.8)	Mucosal melanoma (n = 81)
			Other modalities without immunotherapy (n = 61)		
D’angelo 2017	United States	889	Immunotherapy (n = 889)	7.4 (6.2-–8.6)	Mucosal melanoma (n = 86)
					Cutaenous melanoma (n = 665)
Simeone 2016	Italy	42	Immunotherapy	5.6 mo	Cutaneous melanoma (n = 40)
					Mucosal melanoma (n = 2)
Schaefer 2017	United States	75	Surgery + immunotherapy (n = 21)	32 (2–231 mo)	Mucosal melanoma (n = 75)
			Surgery + immunotherapy + radiotherapy (n = 5)		
Jung 2017	Korea	104	Immunotherapy (n = 104)	7.1 mo (5.9–8.3 mo)	Acral (n = 33)
			Number of previous therapy (not specified)		Mucosal (n = 27)
			1 (n = 41)		Cutaneous (n = 27)
			2 (n = 34)		Uveal (n = 10)
			3 (n = 29)		Unknown (n = 7)
Shoushtari 2016	United States	60	Immunotherapy (n = 60)	15.3 mo	Acral (n = 25)
			Previous systemic therapy (not specified) (n = 51)		Mucosal (n = 35)
Frakes 2015	United States	38	Immunotherapy (n = 6)	Not specified	Mucosal (n = 38)
			Other modalities (n = 32)		
Kirchoff 2016	United States	227	Surgery (n = 53)	Not specified	Mucosal (n = 227)
			Surgery + other modalities (immunotherapy inclusive) (n = 149)		
Wu 2015	Taiwan	31	Immunotherapy + chemotherapy (n = 31)	55 mo (14.7–95.4 mo)	Acral (n = 11)
					Nodular (n = 4)
					Superficial spreading (n = 1)
					Mucosal (n = 10)
					Other (n = 5)
Bakkal 2015	Turkey	10	Surgery + chemoradiotherapy (n = 4)	Not specified	Mucosal (n = 10)
			Surgery + radiotherapy (n = 5)		
			Surgery + chemotherapy + immunotherapy (n = 3)		
Lian 2013	China	189	Surgery (n = 63)	26.8 mo (5.9–53.9 mo)	Mucosal (n = 189)
			Surgery + immunotherapy (n = 63)		
			Surgery + chemotherapy (n = 63)		
Alexander 2014	Australia	104	Immunotherapy (n = 104)	7 mo (0–30 mo)	Cutaneous (n = 79)
					Mucosal (n = 8)
					Uveal (n = 11)
Sun 2013	China	68	Immunotherapy (n = 15)	Not specified	Mucosal (n = 68)
			Chemotherapy (n = 29)		
			Multimodal treatment not specified (n = 37)		
			Radiotherapy (n = 20)		
Vecchio 2014	Italy	71	Previous treatments not specified:	21.8 mo (1.0–32.7 mo)	Mucosal (n = 71)
			1 (n = 47)		
			2 (n = 14)		
			≥3 (n = 10)		
Keller 2013	United States	73	Surgery (n = 26)	27.5 mo	Mucosal (n = 73)
			Surgery + immunotherapy (n = 7)	(0–183 mo)	
			Surgery + chemotherapy(n = 22)		
			Surgery + radiotherapy (n = 18)		
Adenis 2013	United Kingdom	26	Previous treatment modalities (combinations not specified) (n = 26)	Not specified for all cancers	GIST (n = 17)
			Immunotherapy (n = 26)		Chordoma (n = 7)
					Mucosal (n = 2)
Mun 2013	Korea	1	Surgery + immunotherapy + chemotherapy	Not applicable	Mucosal (n = 1)
Sun 2012	China	51	Surgery + immunotherapy ± chemotherapy (n = 11)	Not specified	Mucosal (n = 51)
			Other therapy (not specified) (n = 40)		
Wang 2012	China	61	Immunotherapy + chemotherapy + other unspecified (n = 34)	21.0 mo (5–80 mo)	Mucosal (n = 61)
			Surgery alone (n = 13)		
			Radiotherapy (n = 17)		
Saigal 2012	United States	17	Surgery alone (n = 5)	35.2 mo (5–225 mo)	Mucosal (n = 17)
			Surgery + immunotherapy + other modalities (n = 7)		
			Surgery + other modalities excluding immunotherapy (n = 5)		
Moreno 2010	United States	58	Immunotherapy + other modalities not specified (n = 21)	Not specified	Mucosal (n = 58)
Narasimhan 2009	United States	18	Surgery alone (n = 8)	Not specified	Mucosal (n = 18)
			Surgery + immunotherapy ± other modalities not specified (n = 8)		
			Surgery + other modalities (excluding immunotherapy) (n = 18)		
Bedlikian 2008	United States	616	Chemo therapy ± interferon (n = 352)	Not specified	Skin (n = 497)
			Biochemotherapy (n = 264)		Unknown primary (n = 83)
					Mucosal (n = 21)
					Uveal (n = 15)
Krengli 2006	Italy	74	Surgery (n = 17)	20 mo (1–207 mo)	Mucosal (n = 74)
			Surgery + radiotherapy (n = 42)		
			Radiotherapy (n = 11)		
			Chemoimmunotherapy (n = 4)		
Garzino-Demo 2004	Italy	10	Surgery + immunotherapy + other modalities (n = 8)	Not specified	Mucosal (n = 10)
			Surgery + other modalities (excluding immunotherapy) (n = 2)		
Maxwell 2018	United States	20	Surgery + other modalities (excluding immunotherapy) (n = 10)	9.5 mo (4–24 mo)	Mucosal (n = 21)
			Surgery + chemotherapy + immunotherapy (n = 10)		
Hamid 2018	United States	1567	Immunotherapy + prior modalities not specified (n = 1567)	Not specified	Mucosal (n = 84)
					Non mucosal not specified (n = 1483)
Sayed 2017	United States	72	Surgery + immunotherapy (n = 17)	Not specified	Mucosal (n = 72)
			Surgery + other modalities (excluding immunotherapy) (n = 55)		
Liu 2017	China	51	Immunotherapy ± other modalities (n = 13)	59.0 mo (11–123 mo)	Mucosal (n = 51)
			Other modalities excluding immunotherapy (n = 38)		
Simeone 2017	Italy	42	Immunotherapy ± other therapies (n = 42)	5.6 mo	Cutaneous (n = 40)
					Mucosal (n = 2)
Ascierto 2016	Italy	1	Immunotherapy	Not applicable	Mucosal (n = 1)
Shoushtari 2016	United States	60	Immunotherapy + other modalities (n = 51)	10.6 mo	Mucosal (n = 35)
			Immunotherapy alone (n = 9)		Acral (n = 25)
Frakes 2015	United States	38	Immunotherapy + other modalities (n = 6)	58 mo (7–118 mo)	Mucosal (n = 38)
			Other modalities excluding immunotherapy (n = 32)		
Swegal 2014	United States	25	Immunotherapy + other modalities (n = 6)	20.4 mo (2.4–172 mo)	Mucosal (n = 25)
			Other modalities excluding immunotherapy (n = 19)		
Tajudeen 2014	United States	14	Immunotherapy + other modalities (n = 1)	Not specified	Mucosal (n = 14)
			Other modalities excluding immunotherapy (n = 13)		
Keller 2013	United States	73	Surgery + immunotherapy (n = 22)	27.5 mo (0–183 mo)	Mucosal (n = 73)
			Other modalities excluding immunotherapy (n = 51)		
Krengli 2006	Italy	74	Immunotherapy with chemotherapy (n = 4)	20 mo	Mucosal (n = 74)
			Other modalities excluding immunotherapy (n = 70)		
Wada 2004	Japan	31	Immunotherapy ± other modalities (n = 11)	16 mo (1–214 mo)	Mucosal (n = 31)
Owens 2003	United States	48	Biochemotherapy ± immunotherapy (n = 12)	Not specified	Mucosal (n = 48)
			Other modalities (n = 36)		
Stern 1991	United States	42	Immunotherapy ± chemotherapy (n = 29)	46 mo	Mucosal (n = 42)
			Other modalities excluding immunotherapy (n = 13)		
Kim 2016	Korea	27	Immunotherapy ± other modalities (n = 28)	32.1 mo (24.9–39.1 mo)	Acral (n = 10)
					Mucosal (n = 9)
					Cutaneous (n = 8)
Liao 2014	United States	14	Immunotherapy with other modalities (n = 1)	49 mo	Mucosal (n = 14)
			Other modalities excluding immunotherapy (n = 13)		

## 3. Results

Of the 352 titles and abstracts included in the search, 52 were eligible for final synthesis. The Preferred Reporting Items for Systematic Reviews and Meta-Analyses flowchart (Fig. 1) shows the reasons for exclusion at each level of the screening process.

In the included studies, it was found that there wasn’t a consistency in survival rates, with some papers citing 1-, 2-, 3-year survival rates, others showing 2-, 3-, and 5-year survival rates, and others showing 2-, 5-, and 10-year survival rates. Additionally, not all papers cited survival rates with immunotherapy vs nonimmunotherapy.

We sought to standardize the survival rates at 2, 3, and 5 years. Additionally, we stratified survival rates based on treatment with immunotherapy with or without other modalities and nonimmunotherapy-based treatment, regardless of the chosen modality.

**Figure 2.**
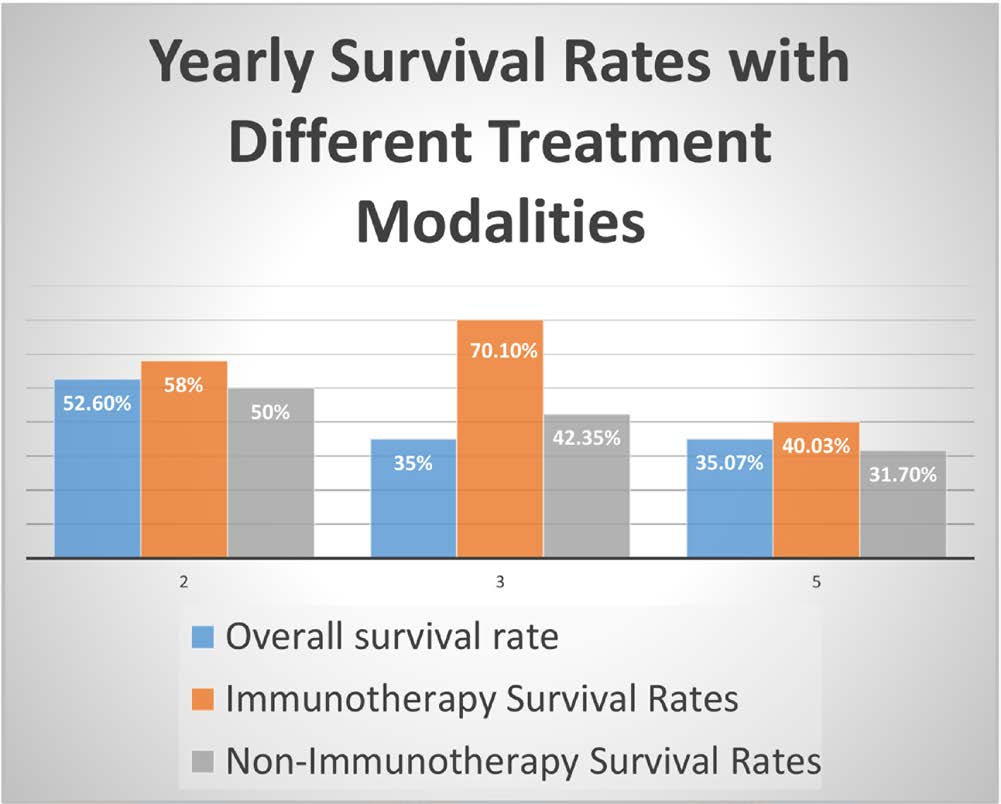
Graph showing comparison of yearly survival rates following different treatment modalities (immunotherapy vs nonimmunotherapy). This figure shows the yearly survival rates (overall), taken as an average of survival rates with and without immunotherapy (shown above). Survival rates chosen for this study were taken at 2-, 3-, and 5-yr follow-up period.

The graph (Fig. 2) demonstrates that survival rates with different treatment modalities spread across 2, 3, and 5 years. The numbers were obtained by gathering data on survival rates in percentages from the different papers at the desired year interval and calculating the median.

At 2 years, the overall survival rate was 52.6%, with treatments including immunotherapy showing a 58% survival rate and treatment without immunotherapy showing a 50% survival rate. Similarly, at 3 years, overall survival was 35%, with 70.1% survival rates in the immunotherapy group and 42.35% in the nonimmunotherapy group. At 5 years, the overall survival was 35.7%, with 40.03% survival in immunotherapy treatment and 31.7% in nonimmunotherapy treatment.

The results of the literature review clearly showed that in the limited database, the involvement of immunotherapy showed better overall survival outcomes.

None of the papers reviewed, however, commented on the quality of life in those who survived at every interval, treatment-related complications, involving significant disabilities, or death in more severe cases.

## 4. Discussion

Mucosal melanomas of the head and neck represent a relatively small pool of malignancies. Not until 2018 was there work by a team of surgeons, medical oncologists, clinical oncologists, radiologists, pathologists, nurses, as well as patients and carer representatives to form clear guidelines on how to manage mucosal melanoma of the head and neck, with a clear pathway diagram outlining steps of referral, assessment and staging, diagnosis, management, and treatment.^[6]^

This paper explores the literature for available studies examining mucosal melanoma of the head and neck and the different treatment modalities available. We searched for treatment modalities consisting of immunotherapy with or without other treatment modalities. We compared these with treatment options that did not involve immunotherapy. The results showed a clear improvement in survival outcomes when immunotherapy was used compared to survival outcomes without immunotherapy at all yearly intervals studied. However, it is pertinent to point out that all studies included a small number of patients, and in many cases, did not clearly define their own inclusion criteria. This could be because the presentation of the disease is variable in terms of both site and duration.

None of the studies reviewed mentioned randomization of patients, which would have eliminated bias and thus decreased likely discrepancies in treatment received, such as the addition of immunotherapy/radiotherapy/chemotherapy to those with poorer prognosis as opposed to surgery alone in those with better prognosis.

There was no report of quality of life in different interventions, and therefore, no subjective feedback on the results of the intervention.

Adjuvant immunotherapy with anti–PD-1 agents following complete resection of high-risk (stage III/IV) melanoma, regardless of subtype, is now the standard of care (NICE Technology Appraisal Guidance TA553 and TA558).^[6]^

Immunotherapy with checkpoint inhibitors has revolutionized the management of melanoma. Ipilimumab, nivolumab, and pembrolizumab are immune checkpoint inhibitors used for the treatment of metastatic melanoma. They activate the immune system to treat melanoma.

Ipilimumab targets cytotoxic T-lymphocyte antigen 4 (CTLA-4). In doing so, it downregulates receptors on activated T cells, whose function is to inhibit T-cell activation. Downregulation of CTLA-4 allows for the expansion of naturally developed melanoma-specific cytotoxic T cells. It resulted in 11% objective response rate and 24% 2-year overall survival.7 The 10-year overall survival of ipilimumab is approximately 22% in a pooled analysis of overall survival data from multiple studies.^[7]^

Nivolumab and pembrolizumab, on the other hand, act by inhibiting programmed cell death ligand-1 (PDL-1). PDL-1 inhibits T-cell proliferation, allowing cancer cells to evade immune surveillance.^[7]^ However, the expression of PDL-1 in mucosal melanomas is not well understood. One study, using immunohistochemical staining in 23 tumor samples from patients with primary mucosal melanoma, found expression in only 13% (3/23) of mucosal melanomas.^[8]^ Treatment outcomes with these modalities have shown mixed results. One study that investigated the outcomes of both mucosal and acral melanoma treatment with PDL-1 inhibitors showed an 11.5% response rate to treatment.^[9]^ D’angelo et al examined the efficacy and safety of nivolumab alone and in combination with ipilimumab in patients with mucosal melanoma. Among patients who received nivolumab, the median progression-free survival was 3.0 months, with an objective response rate of 23.3%. In patients treated with nivolumab in combination with ipilimumab, the median progression-free survival was 5.9 months, with an objective response rate of 37.1%.^[10]^

Wang et al reviewed the effect of Interferon-α-2b as adjuvant therapy and its effect on the prolongation of life in patients with previously resected oral mucosal melanoma.^[11]^ Relapse-free survival was significantly prolonged in patients who received postoperative immunotherapy, but there was no significant difference in overall survival between those who received immunotherapy and those who did not.^[11]^

Frakes et al reviewed a single-center case series of 38 patients, of whom 6 (16%) received adjuvant immunotherapy. The study concluded that immunotherapy was not associated with improvements in local control, progression-free survival, or overall survival.^[12]^

The above-mentioned studies were in contrast to those of Kanetaka et al, who investigated the effect of using lymphokine-activated killer (LAK) cell transfer therapy in mucosal melanoma of the head and neck. The sample size included 13 patients over 18 years of age, with 7 receiving immunotherapy. However, there was no clear explanation as to whether these patients also received chemotherapy. The outcome was that in 7 patients receiving adjunctive LAK cell therapy, the 5-year cause-specific survival rate was 66%, while that in 6 cases without adjunctive LAK therapy was 33%. Although statistical significance was not recognized, LAK therapy has been suggested to improve the prognosis of mucosal melanoma of the head and neck.^[13]^

Long et al conducted a double-blind, placebo-controlled trial, randomizing 870 patients with completely resected stage III melanoma with BRAF mutation to either BRAF-targeted immunotherapy or placebo for 12 months. The rates of distant metastasis-free survival and freedom from relapse were higher than those in the placebo group, with a 53% reduction in relapse or death.^[14]^

In a case report by Studentova et al, following disease progression after surgical resection, the patient was treated with ipilimumab monotherapy that was initially followed by disease progression, but subsequently by disappearance of the primary tumor and overall partial response of the disease 8 months later. However, the effect lasted for only 8 months, and disease progression occurred followed by death 3 months later.^[15]^

A systematic review conducted by Jarrom et al^[16]^ looked at the treatment of mucosal melanoma of the upper airway tract. Eleven studies were selected based on surgery and radiotherapy alone, with no chemotherapy or biological treatment included. Since then, more trials have been conducted on which biologics, including immunotherapy, have been utilized and studied as potential treatment modalities for improving outcomes.

## 5. Conclusion

Immunotherapy outcomes from small studies have provided supporting data for increasing survival rates at yearly intervals in mucosal melanomas of the head and neck.

Larger clinical trials should be performed to accurately distinguish the efficacy and survival outcomes of immunotherapy when compared with treatment modalities, excluding immunotherapy.

The ability to perform larger trials is limited by the rarity of mucosal melanomas of the head and neck.
